# Single Versus Dual Kidney Transplants From Marginal Donors: Balancing Survival and Resource Utilization

**DOI:** 10.1155/joot/7744010

**Published:** 2025-10-25

**Authors:** Kaufman D. M., J. D. Perkins, Bakthavatsalam R., Leca N., Sibulesky L.

**Affiliations:** ^1^Division of Transplant Surgery, Department of Surgery, University of Washington, Seattle, Washington, USA; ^2^Clinical and Bio-Analytics Transplant Laboratory (CBATL), Department of Surgery, University of Washington, Seattle, Washington, USA; ^3^Division of Nephrology, Department of Medicine, University of Washington Medical Center, Seattle, Washington, USA

**Keywords:** dual kidney transplant, kidney graft survival, kidney transplant patient survival, single kidney transplant

## Abstract

**Background:**

With population aging and increasing prevalence of kidney disease, a greater number of older patients could benefit from a kidney transplant. Organ shortage has led to expanding the pool of potential donors, including both kidneys from the same donor as dual transplants into a single recipient. At present, there is no consistent criteria for determining suitability for single versus dual transplant.

**Methods:**

We performed a retrospective analysis of the Organ Procurement and Transplantation Network database of all recipients undergoing single or dual kidney transplants from deceased donors from December 4, 2014, to March 31, 2024, excluding en bloc donors. We examined patient and graft survival rates and graft function in dual versus single kidney transplantation. In addition, we analyzed potential survival differences between using a single kidney transplantation over dual transplantation.

**Results:**

During the study period, there were a total of 1015 dual kidney transplant recipients and 134,933 single kidney recipients. The donors of the dual transplants were older, had higher KDRI, and had increased rates of glomerulosclerosis, with > 20% glomerulosclerosis seen in 20% of the kidneys. Using 3:1 propensity matching, we did not observe a significant difference in overall patient survival. We did observe a significant increase in graft survival with dual transplants. Transplanting all dual kidneys as single kidneys could result in a 0.9% increase in overall successful transplants and a 3% reduction in waitlist deaths.

**Conclusion:**

Careful donor and recipient matching are crucial to optimize outcomes in this population. More emphasis needs to be placed on maximizing survival benefit from each donor kidney.

## 1. Introduction

The population is aging, and there is an increasing prevalence of chronic kidney disease (CKD) and end-stage renal disease (ESRD) that has led to a rising number of older patients requiring kidney transplants [1]. Kidney transplantation offers the best outcomes for patients with ESRD, improving both survival and quality of life compared to dialysis [2]. At the same time, there continues to be a shortage of donors. To close the gap between supply and demand and decrease mortality on the waiting list, more marginal organs are being used, including kidneys from donors with acute kidney injury, DCD kidneys, and kidneys from older donors.

There is increasing evidence that older recipients, even when receiving kidneys from older donors, have significant improvement in survival compared to remaining on dialysis [3]. The analysis by Rao et al. of over 2000 patients aged 70 and older receiving expanded criteria donor kidneys had a significantly lower mortality risk (RR = 0.75; *p* < 0.0001) when compared to those waiting on the transplant list [4]. Arcos et al., studying patients over 60 years old, demonstrated an adjusted risk of death 12 months after receiving a kidney from donors ≥ 80 years to be 0.54 (95% confidence interval, 0.38–0.77; *p* < 0.0001) in comparison with those who remained on dialysis [3].

Furthermore, the outcomes of kidney transplantation in elderly recipients are significantly affected by the number of years they have spent on dialysis prior to the transplant. Haller et al. showed that the rate of death was higher in patients on pretransplant dialysis for > 1.5 years (HR, 1.62; 95% CI (1.43–1.83)) compared with pretransplant dialysis for < 1.5 years [5]. Heldal et al. showed that in recipients 70 years of age and older, one of the factors that was negatively associated with patient survival was time on dialysis before transplantation (HR 1.29 per year, 95% CI (1.06–1.59)) [6].

One of the strategic approaches to maximize the use of available organs is dual kidney transplantation. Dual kidney transplantation, the transplantation of two donor kidneys into a single recipient, is a practice that was introduced in 1996 and developed to increase the use of kidneys from older donors [7]. Currently, there are no consistent criteria for determining suitability for single versus dual transplant.

The aim of the present study was to evaluate the clinical outcomes, including both short-term and long-term patient and graft survival rates and graft function, in dual versus single kidney transplantation, utilizing a national database since the implementation of the kidney allocation system in 2014. In addition, we aimed to analyze potential survival differences of using a single kidney transplantation over dual transplantation from a resource utilization perspective.

## 2. Materials and Methods

All data were from the Organ Procurement and Transplantation Network (OPTN) data released April 15, 2024 for recipients that received a single or dual kidney transplant from a deceased donor from December 4, 2014 to March 31, 2024. The United Network for Organ Sharing (UNOS), as the contractor for the OPTN, supplied this data. The interpretation and reporting of these data are the responsibility of the authors and in no way should be seen as an official policy of or interpretation by the OPTN or the U.S. Government. The University of Washington Human Subjects Division deems the OPTN database de-identified and publicly available and thus, not human subjects' data. Therefore, this study was exempt from human subjects' review and was exempt from approval from an ethics board.

We conducted a retrospective analysis of all ≥ 18-year-old adult U.S. recipients who underwent a single kidney transplant or a dual transplant from a deceased donor with a reported kidney donor risk index (KDRI) value and survival data. Patients receiving pediatric en bloc kidney transplants or kidney transplants from living donors were excluded. We did not exclude patients being re-transplanted. Patient survival, all-cause kidney graft survival, and death-censored kidney graft survival were recorded. Recipient factors included in our analysis included age, sex, race/ethnicity, estimated post-transplant survival score (EPTS), years on dialysis or preemptive transplant, presence of peripheral vascular disease (PVD), etiology of kidney disease, body mass index (BMI), cPRA, immunosuppressive induction therapy (the value of combination induction refers to the practice of using multiple biological agents for induction as recorded in the OPTN dataset), and maintenance immunosuppression at the time of discharge from the transplant hospitalization. Donor factors included in our analysis were age, KDRI, cause of death being CVA, history of hypertension, diabetes mellitus, donation after cardiac death, terminal creatinine, history of cigarette use, method of kidney preservation (static cold storage versus machine perfusion), sex, cold ischemia time in hours, if the kidney was biopsied and percent of glomerulosclerosis, inotropic support of the donor just prior to organ retrieval, sharing, and distance between donor and transplant hospital. ABO incompatibility and HLA mismatch were recorded between recipient and donor. Outcome measures analyzed were length of stay (LOS) in hospital for the transplant procedure, delayed graft function (DGF) as defined by the recipient requiring dialysis in the first week after transplantation, kidney transplant rejection episodes at 6 months and 1 year after transplant, and serum creatinine at 6 months and at 1 year following transplant.

The presence of PVD and inotropic support variables had less than 1.0% missing data, and the majority of “no” was given. BMI was missing in 241 recipients and imputed with linear regression using age, gender, and race/ethnicity. CIT was missing in 203 and imputed with linear regression using type of sharing and distance between donor and transplant hospital. Length of transplant hospital stay was missing in 588 and was imputed with the median value.

### 2.1. Statistical Analysis

Continuous variables are reported as the median and IQR, and categorical variables are presented as counts and percentages. Statistical testing was performed using the Wilcoxon rank-sum test for continuous variables and the chi-square test of independence for categorical variables. Propensity score matching was conducted to balance the variables in the demographic table using the nearest neighbor method with a logit link function to estimate propensity scores. Initially a 1:1 match was performed between dual and single kidney transplants. To increase the sample size while maintaining balance, both a 2:1 and subsequently a 3:1 matching were implemented. The final 3:1 matching was selected as all variables achieved an absolute standardized mean difference within ±0.1, indicating adequate balance. Kaplan–Meier survival analysis with the log-rank test was used to determine survival between dual and single transplants on the propensity match set. Cox proportional hazards models were created to determine the hazard ratios for patient survival, all-cause graft survival, and death-censored graft survival on the propensity-matched set.

**Figure 1:**
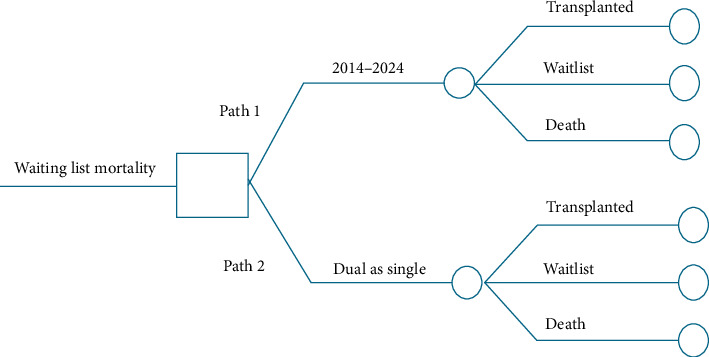
A microsimulation analysis of evaluating the impact of increasing the transplant probabilities by transplanting all dual kidneys as single transplants.

A microsimulation analysis was conducted on the kidney transplant waiting list for 100,000 candidates over an 11-year period to evaluate the impact of increasing the transplant probabilities by transplanting all dual kidneys as single transplants on overall mortality on the waitlist. The probabilities used include those from each year from 2014 to 2024 on being transplanted, having a dual transplant, staying on the waiting list, and death. This allocation course was compared to a course where the small probability of being transplanted increased by converting all dual kidney transplants to single transplants over this time period. Patients were modeled across three states: transplanted, waitlist, and death (Figure 1). Two pathways were compared: Path 1 (current allocation strategy) and Path 2 (a modified strategy with slightly increased transplant probabilities when all dual transplants were transplanted as a single kidney transplant). The goal was to determine whether small adjustments by converting dual kidney transplants into single kidney transplants could lead to improvements in wait list deaths.

All results were considered significant with a *p*-value < 0.05. Statistical analyses were performed using JMP-Pro Version 17.0.0 (SAS Institute, Inc., Cary, NC, USA) and R Core Team (2022), or R: A language and environment for statistical computing. R Foundation for Statistical Computing, Vienna, Austria. URL https://www.R-project.org/. The matchit package in R was used to perform the propensity score matching. The microsimulation analyses were conducted using Python (Version 3.12.8).

## 3. Results

Using the OPTN dataset between December 4, 2014 and March 31, 2024, we identified a total of 1015 dual kidney transplant recipients (excluding en bloc kidney transplants) and 134,933 single kidney recipients (Table 1).

As expected, donor demographics differed, with donors of dual kidneys being significantly older (58 vs. 40 years, *p* < 0.001), having a higher KDRI (1.88 vs. 1.21, *p* < 0.001), and more often showing glomerulosclerosis > 20% (20% vs. 1.1%, *p* < 0.001) (Table 1). Additional donor characteristics that contribute to KDRI also showed consistent differences, with dual kidney donors more frequently having a history of hypertension, diabetes mellitus, and cerebrovascular accident as the cause of death, along with higher terminal creatinine and greater use of cardiac death donors. These differences underscore the higher-risk profile of dual kidney donors, though we note these findings largely reflect the components already incorporated into the KDRI score. Finally, dual kidney transplants were associated with longer cold ischemia times, greater use of machine perfusion, and broader geographic sharing, with correspondingly greater distances between donor and transplant centers (median 137 vs. 90 miles, *p* < 0.001).

Similarly, we observed that recipients of dual kidneys were significantly older (65 vs. 55 years old, *p* < 0.001) with higher EPTS (68 vs. 51, *p* < 0.001), with higher rates of PVD (15% vs. 12%, *p* < 0.001), but fewer years of dialysis (*p* < 0.001). We did observe significantly higher rates of IL-2 receptor antagonists and lower rates of lymphocyte-depleting induction in recipients of dual kidneys (22% vs. 16% and 77% vs. 80% respectively, *p* < 0.001) (Table 1). As expected, these findings are suggestive of selective use of dual kidneys for older patients with anticipated high dialysis mortality and morbidity.

**Figure 2:**
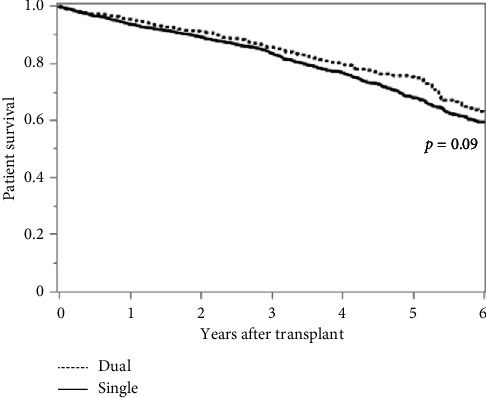
Patient survival between dual and single kidney transplantation.

**Figure 3:**
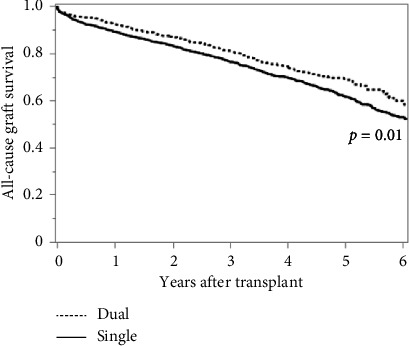
Overall graft survival of dual versus single kidney transplants.

**Figure 4:**
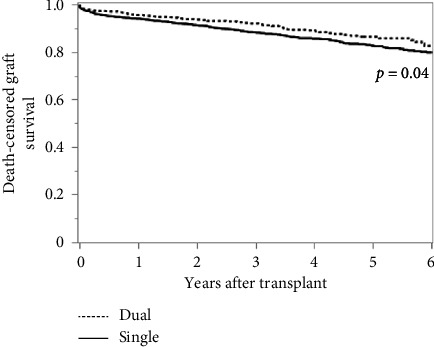
Death-censored graft survival of dual versus single kidney transplants.

Using 3:1 propensity matching (Supporting Table 1), we performed both Kaplan–Meier survival analysis and multivariable Cox proportional hazard models. Based on the log-rank test, we did not observe a significant difference in overall patient survival between dual and single kidney transplantation (Figure 2, Table 2). We did however, observe a significant increase in graft survival with dual kidney transplantation (*p*=0.01) as well as death-censored graft survival (*p*=0.04) (Figures 3 and 4, Tables 3 and 4). Consistent with the Kaplan–Meier survival analysis, we observed a nonsignificantly decreased hazard ratio for patient survival of 0.85 (CI 0.72–1.01: *p*=0.06) with the use of dual kidney transplants. Again, we noted improvement in graft survival, both without and with death censoring (HR 0.79; CI 0.68–0.92 and 0.73; CI 0.58–0.93 with *p*=0.003 and *p*=0.01, respectively) (Table 5).These findings demonstrate that despite the anticipated worse performance of the individual kidneys used for dual kidney transplant, the use of both kidneys does in fact produce increased graft survival relative to matched single kidneys; however, this difference did not produce a meaningful improvement in patient survival.

Given the increased technical complexity with dual kidney transplantation, we assessed LOS for the index hospitalization following transplantation (Table 6). LOS was significantly longer with the use of dual kidneys (*p* < 0.001). The DGF rate was also found to be higher, 39.3% in the dual kidney group versus 32.7% in the single kidney group (*p* < 0.001). To further evaluate the causes of increased graft survival with the use of dual kidneys, we examined rejection rates at 6 months and 1 year post-transplantation but did not find a significant difference at either time point. Kidney function was better at 6 months and 1 year in the dual transplant group, with Cr 1.2 mg/dL in the dual transplant group versus 1.6 mg/dL in the single transplant group (*p* < 0.001) at 1 year. The eGFR at 1 year in the dual transplant group had a median of 51.9 (36.9–69.5), and the single transplant group had a median of 38.7 (28.8–52.6) (< 0.001).

A subanalysis on death-censored graft survival using 2023 data for transplant volumes (18,873 single and 184 dual) and survival results for the Cox Model on the propensity dataset, transplanting all dual kidneys as single kidneys could result in 140 (135–146) additional successful transplants in 5 years, representing a 0.9% increase in overall successful transplants.

Using microsimulation analysis, we have found that if all kidneys that were transplanted as duals were transplanted as single kidney transplants, there would be a 3% reduction in waitlist deaths.

## 4. Discussion

Close to 90,000 patients are waiting for a kidney transplant, with 20% of them being aged 65 years or older. The likelihood of elderly patients being listed and receiving a transplant is low [8]. Only about 8% of listed patients aged 65–74.9 years receive a transplant within 5 years. Close to half are projected to die while waiting on the transplant list [9]. With timely transplantation and with fewer years on dialysis, older patients do derive a survival benefit over remaining on dialysis [3, 4, 10]. Rose et al. demonstrated that patient survival was lower than death-censored allograft survival among recipients aged 60 years and older receiving kidney grafts from deceased donors ≥ 65 years old, indicating that these kidneys provided a lifetime of function for this patient age group [11]. Savoye et al. demonstrated that patients aged 60 years and over who did not undergo transplantation had an adjusted risk of death 2.31 times higher than that of transplanted patients of the same age group (*p* < 0.0001) when receiving a graft from expanded criteria donors [12]. Despite these findings, greater than 60% of kidneys from donors ≥ 65 years are not placed [13].

Dual kidney transplantation often becomes an option when the kidneys are declined to be transplanted as single transplants. Dual kidney transplantation, defined as transplantation of 2 kidneys from the same donor to a single recipient, has been developed to increase the use of the kidneys from the older donors with marginal kidneys, the kidneys that potentially otherwise would not be used [7]. Currently, there are no widely accepted defined donor criteria to guide clinicians when choosing kidneys for transplantation, either as single versus dual.

There are various protocols that guide the decision-making of which kidneys could be transplanted as single versus dual grafts. Remuzzi et al. introduced a biopsy-based scoring system for kidneys, with scores ranging from a minimum of 0 (indicating the absence of renal lesions) to a maximum of 12 (indicating the presence of marked changes in the renal parenchyma) [14]. Andres et al. proposed single kidney transplantation from donors between 60 and 74 years old with glomerulosclerosis < 15% and double transplantation from donors aged from 60 to 74 with a percentage of glomerulosclerosis of 15% or more [15].

Our analysis of the U.S. national database, starting in 2014 with a new KAS, demonstrated that the mean age of the donors of the dual kidneys was 58 years old with a mean KDRI of 1.88. These donors had a mean terminal Cr of 1.12 mg/dL, and just under 50% had a glomerulosclerosis of equal to or less than 10%. The average cold ischemia time was 23 h.

In our propensity-matched analysis of the dual kidney transplants and single transplants, we observed an improved overall and death-censored graft survival in a dual transplant group. Patient survival was not statistically different in those receiving dual versus single kidneys. Kidney function at 1 year post-transplant was statistically better in the dual transplant group compared to the single transplant group, 1.37 ± 0.6 and 1.70 ± 0.77 mg/dL, respectively. Upon analyzing our data, we found that while the difference in outcomes between the groups reached statistical significance, the magnitude of the difference may not be clinically significant, as small improvements in creatinine with dual kidney transplantation likely do not lead to symptomatic improvement, raising a question of the optimal use of these kidneys. Furthermore, if dual kidney transplant is utilized selectively in older patients to improve access to transplantation, the impact of long-term benefits such as mildly improved graft survival may have limited benefit in these recipients.

Similar findings were noted in other studies. Ibrahim et al. in the United Kingdom reported on dual versus single kidney transplant outcomes. In this study the donors of dual organs were older and had higher KDRI when compared to single kidney transplants (median, 73 vs. 66 years; *p* < 0.001) and higher United States Kidney Donor Risk Indices (2.48 vs. 1.98; *p* < 0.001), respectively. After adjusting for confounders, they found no difference in 5-year graft survival (hazard ratio, 0.81; 95% CI, 0.59–1.12) and no difference in patient survival. Graft function was significantly better at 1 year in the dual kidney group. They also found that at 3 years, of 194 patients who received dual kidney transplants, 47 (24%) had an eGFR of ≥ 60 mL/min per 1.73 m^2^, suggesting these kidneys could potentially have been successfully implanted as single kidney transplants into two recipients [16].

Lee et al. in Korea reported on the utilization of deceased donors greater than 70 years old with one of two risk factors: serum creatinine level over 3.0 mg/dL or eGFR under 30 mL/min. Death-censored graft survival 3 years after KT was 95.9% in the ECD group and 100% in the dual kidney transplant group. At 3 years, patient survival was 96.2% in the ECD group and 100% in the DKT group. The DGF rate was similar, and there was no statistical difference on eGFR at 3 years [17].

In evaluating dual kidney transplantation for older patients, it is essential to consider their reduced capacity to have complications. Older patients often have multiple comorbidities and diminished physiological reserves. Furthermore, older recipients may have less access to transplantation despite the survival benefits of kidney transplantation [10, 18]. We observed that the LOS was significantly longer in patients receiving dual kidney transplants, likely reflecting the slower recovery associated with the increased complexity of the surgical procedure. The operating times are longer. There also appears to be a higher incidence of complications. Rigotti reported on the complications of the unilateral placement of two grafts compared to bilateral placement. Complications noted were renal vein thrombosis, wound dehiscence, and lymphocele. While more frequent in the unilateral group, it did not reach statistical significance [19]. Mendel et al. reported on a total of 19/39 (48.7%) surgical revisions in the DKT group versus 52/155 (33.6%) in the SKT group (*p*=0.0788). The rate of venous thrombosis was statistically higher in the DKT group (*n* = 5; 12.8%) compared to the SKT group (*n* = 5; 3.2%; *p*=0.0154) [20].

In our analysis, we also demonstrated that the DGF rate was higher in the dual transplant group compared to the single transplant (39.3% vs. 31.7%). The LOS was found to be statistically longer in the dual group as well.

Based on our subanalysis using our propensity results, transplanting all dual kidneys as single kidneys could result in 140 (135–146) additional successful transplants in 5 years for each year of transplants, representing a 0.9% increase in overall successful transplants. Our simulation analysis revealed a 3% reduction in cumulative deaths on the waiting list for each year.

We acknowledge that our analysis has limitations. It is based on data from a national database, which may be subject to inherent selection biases and missing variables. Additionally, the criteria used for organ selection in transplantation are not explicitly detailed, potentially limiting our ability to account for confounding factors. However, our use of propensity matching mitigates some of these confounding effects, aligning with best practices in transplantation research. An additional limitation is the duration of follow-up. Considering the observed trends toward improved survival at later time points, it is possible that longer-term follow-up could demonstrate more pronounced differences in outcomes with dual kidney transplantation.

From the perspective of kidney utilization, it is unclear what the impacts of dual versus single kidney transplantation may produce. An unclear area would be the utilization of discordant kidneys with differing biopsy results or pump numbers, where the second kidney would be unlikely to be utilized if it were not transplanted as a dual organ or would be unlikely to be utilized due to scheduling limitations.

Successful transplantation of the older donor kidneys needs careful matching of the theoretical number of nephrons in the transplant with the metabolic demands of the recipient. One of the main challenges is how best to assess a graft's quality prior to its implantation. While there might be a role for dual kidney transplantation, based on our analysis, we believe a significant number of kidneys transplanted as duals could have been successfully transplanted as single organs without compromising patient survival and benefitting more patients on the waiting list, despite the marginally improved graft outcomes with the use of dual kidney transplantation.

In conclusion, one of the main challenges is how best to assess a graft's quality prior to its implantation, and thus, there are ongoing uncertainties regarding identifying kidneys suitable for single kidney versus dual kidney transplant. There is still a need for more studies to find the best allocation criteria that would permit transplantation to the highest number of patients. Careful donor and recipient matching are crucial to optimize outcomes in this population. More emphasis needs to be placed on maximizing survival benefit from each donor kidney.

## Figures and Tables

**Table 1:** Demographic table of dual versus single kidney transplants from December 4, 2014, to March 31, 2024, in the United States.

Characteristic	Overall, *N* = 135,948^1^	Dual, *N* = 10,15^1^	Single, *N* = 134,933^1^	*p* value^2^
*Donor*				
Age, years	40 (29, 52)	58 (50, 65)	40 (29, 51)	< 0.001
Pump vs. ice preservation	76,188 (56%)	736 (73%)	75,452 (56%)	< 0.001
KDRI^3^	1.21 (0.99, 1.50)	1.88 (1.56, 2.23)	1.21 (0.99, 1.50)	< 0.001
Cause of death: CVA	30,864 (22.7%)	423 (41.7%)	30,441 (22.6%)	< 0.001
History of hypertension	39,931 (29.4%)	625 (61.6%)	39,306 (29.1%)	< 0.001
Diabetes mellitus	11,376 (8.4%)	236 (23.3%)	11,140 (8.3%)	< 0.001
Donation after cardiac death	38,769 (28.5%)	323 (31.8%)	38,446 (28.5%)	0.02
Terminal creatinine (mg/dL)	0.93 (0.70–1.42)	1.12 (0.77–1.80)	0.93 (0.70–1.42)	< 0.001
Cigarette use	27,905 (20.5%)	301 (29.7%)	27,604 (20.5%)	< 0.001
Female	51,026 (38%)	540 (53%)	50,486 (37%)	< 0.001
Cold ischemia time (h)	18 (13, 24)	23 (17, 29)	18 (13, 23)	< 0.001
Sharing				< 0.001
Local	79,303 (58.3%)	480 (47.3%)	78,823 (58.4%)	
Regional	25,718 (18.9%)	220 (21.7%)	25,498 (18.9%)	
National	30,927 (22.7%)	315 (31.0%)	30,612 (22.7%)	
Distance from donor to transplant center, miles	90 (15–211)	137 (29–395)	90 (15–211)	< 0.001
Percent glomerulosclerosis				< 0.001
0%–5%	50,598 (37%)	264 (26%)	50,334 (37%)	
6%–10%	10,410 (7.7%)	157 (15%)	10,253 (7.6%)	
11%–15%	3985 (2.9%)	126 (12%)	3859 (2.9%)	
16%–20%	1789 (1.3%)	102 (10%)	1687 (1.3%)	
20%+	1645 (1.2%)	198 (20%)	1447 (1.1%)	
No biopsy	67,521 (50%)	168 (17%)	67,353 (50%)	
Inotropic support	44,063 (32%)	376 (37%)	43,687 (32%)	0.002

*Recipient*				
Age, years	55 (44, 64)	65 (59, 70)	55 (44, 64)	< 0.001
EPTS^4^	51 (22, 78)	68 (47, 85)	51 (21, 78)	< 0.001
Years on dialysis	3.9 (1.6, 6.3)	2.0 (0.7, 3.6)	3.9 (1.6, 6.3)	< 0.001
Dialysis groups by year				< 0.001
Preemptive	19,717 (15%)	201 (20%)	19,516 (14%)	
< 1 year	6538 (4.8%)	107 (11%)	6431 (4.8%)	
≥ 1 < 5 years	58,821 (43%)	586 (58%)	58,235 (43%)	
≥ 5 < 10 years	42,582 (31%)	106 (10%)	42,476 (31%)	
≥ 10 years	8290 (6.1%)	15 (1.5%)	8275 (6.1%)	
Peripheral vascular disease	15,882 (12%)	156 (15%)	15,726 (12%)	< 0.001
Renal diagnosis				< 0.001
Diabetes mellitus	41,059 (30%)	437 (43%)	40,622 (30%)	
Glomerular	24,722 (18%)	143 (14%)	24,579 (18%)	
Hypertensive	32,145 (24%)	251 (25%)	31,894 (24%)	
Other	18,007 (13%)	105 (10%)	17,902 (13%)	
Polycystic renal disease	9284 (6.8%)	70 (6.9%)	9214 (6.8%)	
Retransplant	10,731 (7.9%)	9 (0.9%)	10,722 (7.9%)	
BMI	28.1 (24.4, 32.3)	27.4 (24.2, 31.3)	28.1 (24.4, 32.3)	< 0.001
cPRA	0 (0, 54)	0 (0, 2)	0 (0, 54)	< 0.001

*Transplant*				
ABO incompatible	2799 (2.1%)	13 (1.3%)	2786 (2.1%)	0.08
HLA mismatch				< 0.001
0	6728 (4.9%)	7 (0.7%)	6721 (5.0%)	
1	1897 (1.4%)	9 (0.9%)	1888 (1.4%)	
2	6737 (5.0%)	26 (2.6%)	6711 (5.0%)	
3	19,529 (14%)	109 (11%)	19,420 (14%)	
4	37,504 (28%)	236 (23%)	37,268 (28%)	
5	43,555 (32%)	398 (39%)	43,157 (32%)	
6	19,998 (15%)	230 (23%)	19,768 (15%)	
Induction immuno				
Lymphocyte depletion	108,174 (80%)	782 (77%)	107,392 (80%)	0.045
IL2RA	22,446 (17%)	223 (22%)	22,223 (16%)	< 0.001
Combination	4238 (3.1%)	49 (4.8%)	4189 (3.1%)	0.002
Steroids	95,344 (70%)	647 (64%)	94,697 (70%)	< 0.001
Maintenance immuno				
Steroids	98,418 (72%)	759 (75%)	97,659 (72%)	0.09
MMF	77,350 (57%)	573 (56%)	76,777 (57%)	0.80
Tacrolimus	128,707 (95%)	923 (91%)	127,784 (95%)	< 0.001

^1^Median (IQR); *n* (%).

^2^Wilcoxon rank sum test; Pearson's chi-squared test.

^3^Kidney donor risk index.

^4^Estimated post-transplant survival.

**Table 2:** Patient survival between dual and single kidney transplantation.

Year	Dual		Single		*p* value
	%	*N*	%	*N*	
0		1015		3045	
1	95.2	709	93.6	2091	0.13
2	90.9	528	89.1	1579	0.16
4	80.0	275	76.6	736	0.10
6	62.3	87	59.4	271	0.06

**Table 3:** Overall graft survival of dual versus single kidney transplants.

Year	Dual		Single		*p* value
	%	*N*	%	*N*	
0		1015		3045	
1	92.2	695	89.2	2031	0.03
2	86.9	513	83.2	1521	0.01
4	74.5	263	69.7	695	0.01
6	59.1	78	52.9	250	< 0.001

**Table 4:** Death-censored graft survival of dual versus single kidney transplants.

Year	Dual		Single		*p* value
	%	*N*	%	*N*	
0		1015		3045	
1	95.6	695	94.1	2031	0.37
2	93.7	513	91.4	1521	0.10
4	89.2	263	85.7	695	0.051
6	82.7	78	79.9	250	0.01

**Table 5:** Cox proportional hazard analysis for patient survival and all-cause and death-censored graft survival.

	Univariable		*p*	Multivariable^∗^		*p*
	HR	CI		HR	CI	
Patient survival						
Dual vs. single	0.86	0.73–1.02	0.09	0.85	0.72–1.01	0.06
All-cause graft survival						
Dual vs. single	0.82	0.70–0.95	0.01	0.79	0.68–0.92	0.003
Death-censored graft survival						
Dual vs. single	0.78	0.62–0.99	0.04	0.73	0.58–0.93	0.01

^∗^Controlled for donor factors of pump vs. ice preservation, KDRI, female, CIT, % glomerulosclerosis, inotropic support, and recipient factors of EPTS, dialysis groups by year, peripheral vascular disease, renal diagnosis, BMI, cPRA, and transplant factors of ABO incompatible, HLA mismatches, and immunosuppressive therapy.

**Table 6:** Outcomes on propensity match set for dual versus single kidney transplants.

Outcomes	Dual *N* = 1015	Single *N* = 3045	*p* value^2^
LOS, days	5 (4–8)	5 (4–7)	< 0.001
Delayed graft function	399 (39.3%)	964 (31.7%)	< 0.001
Rejection 6 months^1^	28/727 (3.9%)	122/2242 (5.4%)	0.10
Rejection 1 year^1^	35/662 (5.3%)	148/1968 (7.5%)	0.052
Creatinine 6 months^1^	1.3 (1.0–1.6) (*N* = 854)	1.5 (1.2–1.9) (*N* = 2575)	< 0.001
eGFR 6 months	52.5 (37.5–71.5)	40.6 (29.6–54.4)	< 0.001
Creatinine 1 year^1^	1.2 (1.0–1.6) (*N* = 752)	1.6 (1.3–2.0) (*N* = 2242)	< 0.001
eGFR 1 year	51.9 (36.9–69.5)	38.7 (28.8–52.6)	< 0.001

^1^Different numbers in analysis due to all patients not having the same time of follow-up.

^2^Wilcoxon rank sum test; Pearson's chi-squared test.

## Data Availability

All data were from the Organ Procurement and Transplantation Network (OPTN) data released on April 15, 2024, for recipients that received a single or dual kidney transplant from a deceased donor from December 4, 2014, to March 31, 2024. The United Network for Organ Sharing (UNOS), as the contractor for the OPTN, supplied these data.

## Nomenclature

KDRI: Kidney donor risk index
UNOS: United Network for Organ Sharing
OPTN: Organ Procurement and Transplantation Network
cPRA: Calculated panel reactive antibody
HLA: Human leukocyte antigen
PVD: Peripheral vascular disease
LOS: Length of stay
DGF: Delayed graft function
CKD: Chronic kidney disease
EPTS: Estimated post-transplant survival

## Supporting Information

Supporting Table: Demographic table of dual versus single kidney transplants using 3:1 propensity matching.
