# Supplementary material for: Single Versus Dual Kidney Transplants From Marginal Donors: Balancing Survival and Resource Utilization
Source: J Transplant. 2025 Oct 25;2025:7744010. doi: 10.1155/joot/7744010 (PMC12579562; doi:10.1155/joot/7744010)
Supplement: Supporting Information — Additional supporting information can be found online in the Supporting Information section. [file 7744010.f1.docx]

**Supplementary Table 1**

| ^1^ Median (IQR); n (%) |
| --- |
| ^2^ Absolute Standardized Mean Differences; values between ±0.1 show good matching |
| ^3^ Kidney Donor Risk Index |
| ^4^ Estimated Post Transplant Survival |
